# Clustering-Based Dual Deep Learning Architecture for Detecting Red Blood Cells in Malaria Diagnostic Smears

**DOI:** 10.1109/JBHI.2020.3034863

**Published:** 2020-10-29

**Authors:** Yasmin M. Kassim, Kannappan Palaniappan, Feng Yang, Mahdieh Poostchi, Nila Palaniappan, Richard J Maude, Sameer Antani, Stefan Jaeger

**Affiliations:** 1 Lister Hill National Center for Biomedical CommunicationsNational Library of Medicine 10952 Bethesda MD 20894 USA; 2 EECS DepartmentUniversity of Missouri-Columbia 14716 Columbia MO 65211 USA; 3 School of MedicineUniversity of Missouri-Kansas City 12273 Kansas City MO 64110 USA; 4 Mahidol-Oxford Tropical Medicine Research UnitMahidol University 26687 Bangkok 10400 Thailand; 5 Centre for Tropical Medicine and Global Health, Nuffield Department of MedicineUniversity of Oxford 6396 Oxford OX3 7LG U.K.; 6 Harvard TH Chan School of Public HealthHarvard University 1857 Boston MA 02115 USA

**Keywords:** Red blood cells (RBCs), white blood cells (WBCs), deep learning, faster R-CNN, connected components, semantic segmentation, superpixel, U-Net

## Abstract

Computer-assisted algorithms have become a mainstay of biomedical applications to improve accuracy and reproducibility of repetitive tasks like manual segmentation and annotation. We propose a novel pipeline for red blood cell detection and counting in thin blood smear microscopy images, named RBCNet, using a dual deep learning architecture. RBCNet consists of a U-Net first stage for *cell-cluster* or superpixel segmentation, followed by a second refinement stage Faster R-CNN for detecting small cell objects within the connected component clusters. RBCNet uses cell clustering instead of region proposals, which is robust to cell fragmentation, is highly scalable for detecting small objects or fine scale morphological structures in very large images, can be trained using non-overlapping tiles, and during inference is adaptive to the scale of cell-clusters with a low memory footprint. We tested our method on an archived collection of human malaria smears with nearly 200,000 labeled cells across 965 images from 193 patients, acquired in Bangladesh, with each patient contributing five images. Cell detection accuracy using RBCNet was higher than 97$\%$. The novel dual cascade RBCNet architecture provides more accurate cell detections because the foreground cell-cluster masks from U-Net adaptively guide the detection stage, resulting in a notably higher true positive and lower false alarm rates, compared to traditional and other deep learning methods. The RBCNet pipeline implements a crucial step towards automated malaria diagnosis.

## I. Introduction

Malaria is a leading cause of death worldwide [1]. The parasitic infectious disease can be transmitted easily to human through mosquito bites that result in over 200 million infections and 400 thousand deaths every year, with children under five accounting for the majority of all malaria deaths worldwide. Although the highest risk region is sub-Saharan Africa, half the world's population is at risk. The disease begins with common cold symptoms such as fever, headache, and chills; which if not treated, may lead to severe complications such as kidney failure, anemia, pulmonary edema, abnormal liver function, cerebral malaria, neuro-disability, seizures, and ultimately death.

Millions of blood smears are examined for malaria parasites by microscopists every year to determine whether a person is infected or not [1], [2]. This procedure requires several steps, beginning with collecting blood smears, staining them and examining the slides to identify different cells and observe infected ones. Manual counting and detection is tedious, costly, slow, and depends on the skills and expertise of the microscopist. Automated algorithms based on machine learning and image processing have the potential to provide fast, cheap, and reliable malaria diagnosis, avoiding erroneous detections that usually occur with manual examination. Recently, convolutional neural networks have become very popular for solving problems in machine learning and computer vision [3]–[5] as the model learns and computes distinctive features from the data without any human intervention. However, these so-called deep learning techniques (DL) need a large amount of annotated data and processing power to learn the weights to produce a predictive model. In the medical field, obtaining labeled data is a bottleneck because it requires expert knowledge. Specifically, for malaria screening and diagnosis, developing accurate automatic blood cell detection is particularly challenging. Different blood smear images can vary in staining, resolution, illumination, cell shape, appearance, color, contrast and debris. Furthermore, cells can clump together, making identification of individual cells harder, and staining artifacts can confuse fragile image analysis methods. Nevertheless, several algorithms and techniques [6] have been developed with the goal to replace manual diagnosis, decrease cost, and speed up diagnosis.

### A. Related Work

Most malaria screening and diagnosis algorithms [6] begin with finding the foreground masks using simple thresholding methods such as Otsu's method [7]–[10], k-means [11], [12], adaptive histogram thresholding [13]–[15], or Zack thresholding [16], followed by different techniques to separate touching cells, which is arguably the main challenge in cell detection. Among those methods are distance transform, watershed, morphological operations, and active contours. Watershed [17] and Active contours [18], [19] are considered superior traditional techniques for cell detection. Although CNNs are now very popular and robust in solving various biomedical problems [20]–[25], there has not been as much work reported for red blood cell (RBC) detection. Most of the existing methods are for classification between different types of cell images [26]–[28] or between infected and uninfected cells, while the core step for detecting and counting cells still depends upon traditional methods. In Liang *et al.* [29], the classification of infected and uninfected cells relies on a convolutional neural network; however, Active contour [18], [19] is used to segment the cells. In Dong *et al.* [30], the authors studied automatic identification of malaria infected cells using deep learning methods, but they segmented images using thresholding techniques and morphological operations, while their cell separation depends upon Hough Circle transform. In Bibin *et al.* [31], the authors propose a deep belief network (DBN) to differentiate between infected and uninfected cells, using a level set method to segment stained objects [32]. In Gopakumar *et al.* [33], the authors present an automated CNN-based framework to classify a region around the candidate locations as either infected or healthy. Those candidate locations are found by thresholding operations, specifically by looking only at regional intensity minima since stained parasite regions usually appear darker than other pixels. In Rajaraman *et al.* [34], the authors use a level-set based algorithm to detect and segment the RBCs, and then use several pre-trained Convolutional Neural Networks (CNN) to classify parasitized and uninfected cells. In Loganathan *et al.* [35], the authors use the entropy estimation method to detect RBCs, then separate the touching cells using distance transform and random walk algorithm, followed by diseased RBCs classification by a deep CNN architecture. In Molina-Cabello *et al.* [36], the authors use Hough Circle transform for cell detection and artificial neural networks for classification as either RBC or not RBC.

There are a few recent papers in the literature that use CNN for RBC detection. In Faliu *et al.* [37], the authors present two models to extract RBCs from holographic images based on convolutional neural networks. However, in the inference stage, the trained model was used to predict each pixel in the image which is computationally expensive and time consuming for large image sizes. In addition, for their best model, they also use internal markers from watershed algorithm to separate the cells. In Yang *et al.* [38], the authors detect and classify cells using Faster R-CNN [39]. They use microscopic images with size equal to $659\times 493$ which is considered the ideal size as input for Faster R-CNN network. However, their images have a small number of cells, while in our paper, the image size is $5312\times 2988$ with dense cells. In Hung. *et al.* [40], the authors use Faster R-CNN for detecting the cells; nevertheless, it lacks important details. The authors mention that the network is trained by cropping tiles with size $448\times 448$, but it is not clear how they test their trained model and whether they get the prediction on the full images or tiles. The authors trained their network using only four patients and tested only on one patient. Compared to their work, in our present paper, we train with 33 patients and test with 193 patients. This manuscript is an extended version of a brief abstract which has preliminary results for few experiments published in [41].

### B. Contribution and Novelty

In our study, we present RBCNet, a novel algorithm based on a dual deep-network architecture to segment cells: U-Net  [42] first separates touching cells and cell clusters in the produced binary mask, followed by Faster R-CNN [39] performing the final cell detection. The novelty behind combining these two well known deep learning networks is in detecting highly overlapped RBCs in large blood smear images. In particular, our approach is scalable to large blood smear images ($5312\times 2988$ pixels) featuring high cell densities, where cells are relatively small objects compared to the overall image size. We train our dual deep-network architecture with image tiles to ensure fast training with a reasonable number of cells in each tile and relatively large tile size to cell size ratio, while presenting connected components to the network instead of regular tiles in the testing stage avoids cutting off cells. Detecting small objects in large images is an ongoing area of research and our study could be a robust solution that eliminates the limitations of the regular strategy which is problematic for cells residing on tile boundaries that usually affect the overall accuracy, as discussed in detail in Section III. Additionally, we compare our work with 11 methods (traditional and deep learning) with several combinations and testing strategies. We have made the RBCNet code available here: https://github.com/nlm-malaria/RBCNet

### C. Motivation, Challenges, and Proposed Pipeline

There are several challenges that motivated us to build our dual pipeline: Our image size is large $5312\times 2988$ compared to the relatively small cell size; further, microscopy smears vary in cell shape, density, illumination, and color. Applying Faster R-CNN or any other object detection network directly to an entire image is computationally expensive, especially with recent object detection networks that have multiple training stages as Faster R-CNN. Object detection networks are usually applied to images for which the shortest side is around 600 pixels, whereas the shortest side in our images is 2988.

**Fig. 1.**
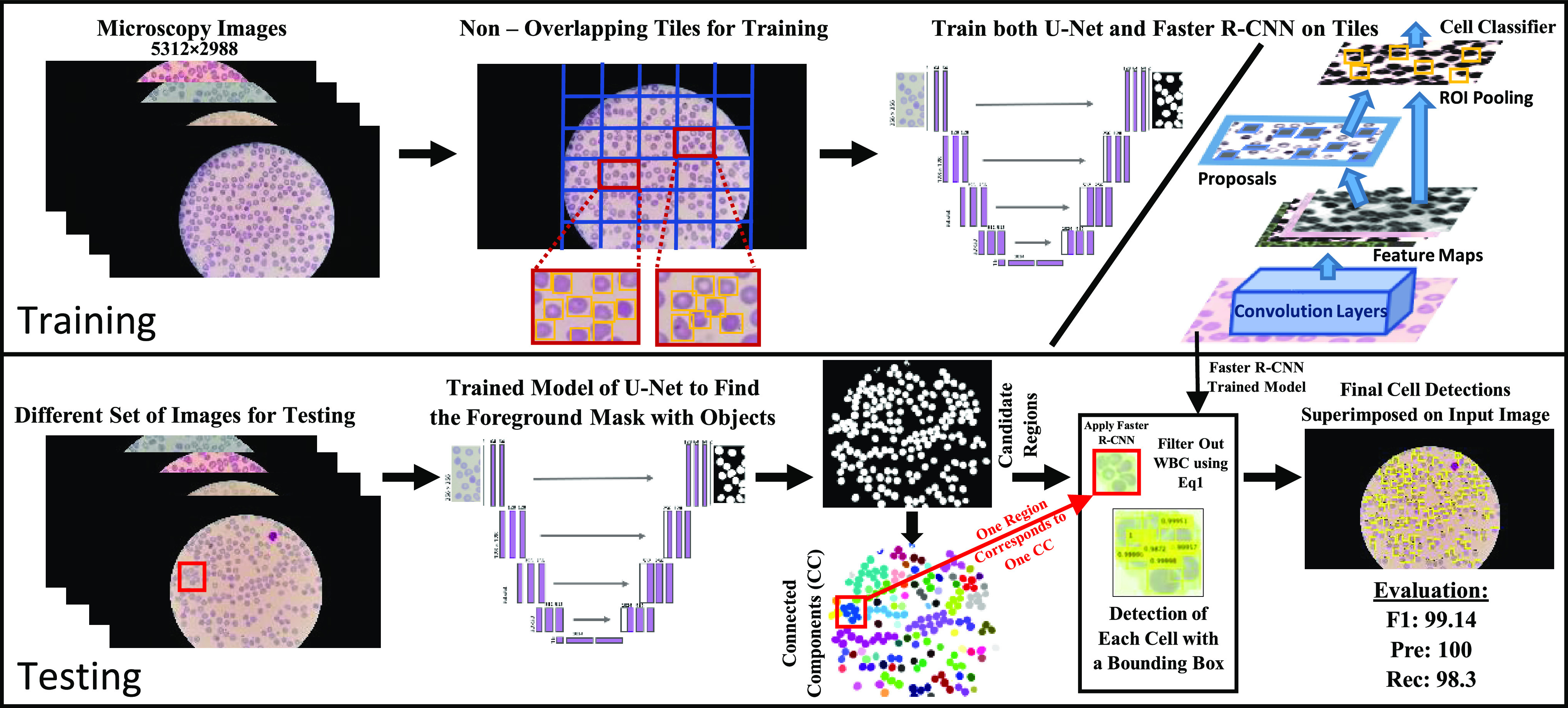
Visualization of our RBCNet pipeline with two stages: training and testing. In the training stage, U-Net and Faster R-CNN are used to train on raw image tiles, while in the inference stage, we apply a Faster R-CNN trained network to cell cluster raw image regions corresponding to the connected components produced by U-Net.

We have developed a novel dual deep architecture based cell segmentation pipeline for segmenting a dense set of RBCs in large thin smear microscopy images as part of an end-to-end fieldable system for rapid fully automated malaria diagnosis [43]. The RBCNet pipeline shown in Fig. 1 consists of two stages including a U-Net architecture with connected component labeling for detecting and extracting cell cluster foreground masks. The second stage uses a Faster R-CNN architecture for refining the cell clusters into individual cells with accurate boundaries. The two stage cascade architecture is individually trained using malaria thin smear microscopy imagery as described in Section II. The key idea is that U-Net architecture guides the detection process in the inference stage by providing robust candidates (clusters of cells) as input to a Faster R-CNN network rather than the standard way of tiling the image. Our pipeline is a robust solution to various challenges imposed by large size images with dense objects.

## II. Malaria Data Set and Methodology

**Dataset:** We tested our proposed pipeline for RBC cell detection with archived thin blood smear images from human patients acquired from Chittagong Medical College Hospital in Bangladesh. Giemsa-stained thin-blood smear slides were collected from *Plasmodium falciparum* infected patients and healthy controls and photographed using a smartphone camera. The slide images were manually annotated by an expert, de-identified, and archived. The Institutional Review Board (IRB) at the National Library of Medicine (NLM), National Institutes of Health (NIH) granted approval to carry out the study within its facilities (IRB$\#$12972). We publish the data here: ftp://lhcftp.nlm.nih.gov/Open-Access-Datasets/Malaria/NIH-NLM-ThinBloodSmearsPf/

**Fig. 2.**
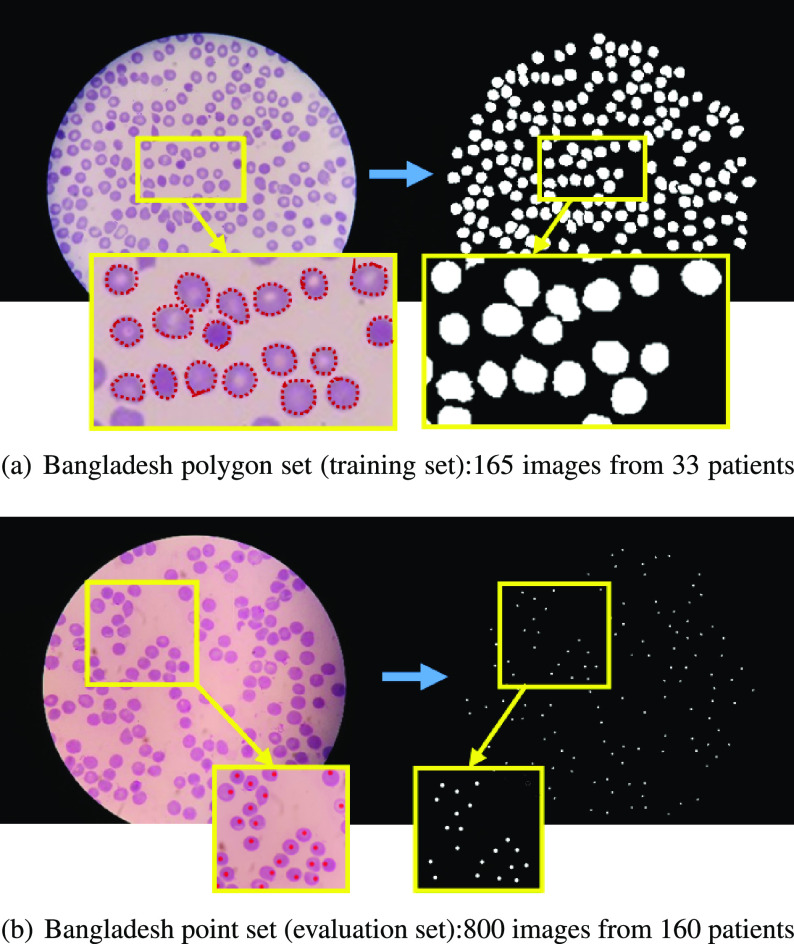
Our Bangladesh data set is divided into two sets according to annotation scheme: (a) Polygon set, cell outlines annotated by polygons for network training, contains 165 images from 33 patients, each patient contributes with 5 images, total number of RBCs is 34,213. (b) Point set, annotated by placing a dot on each cell, used for network evaluation, contains 800 images from 160 patients, each patient contributes with 5 images, total number of RBCs is 162,450.

All images have three color channels with image dimensions of $5312 \times 2988$ pixels. Because images are captured through the eyepiece of the microscope, the visual region is circular. We divided the data set into two parts: a polygon set and a point set. The difference between these two sets lies in the annotation method. In the polygon set, all red blood cells and white blood cells (WBC) have been outlined manually with polygons using the Firefly annotation tool [44],^1^^1^http://www.firefly.cs.missouri.edu. whereas in the point set, cells have been marked by placing a point on each cell, as illustrated in Figure 2. We use the polygon set for training and the point set for evaluation. The polygon set consists of 165 blood smear images from 33 patients, with each patient contributing five slides. The point set consists of 800 images from 160 patients. The total number of RBCs is 34,213 and 162,450 in the polygon and point set, respectively.

**RBCNet architecture and training step:** RBCNet consists of U-Net [42] and Faster R-CNN [39]. U-Net [42] is one type of semantic segmentation networks which means pixel-wise labeling so that each pixel in the image has a unique class or category. Most existing networks classify objects such as cars, people, airplanes, etc. However, U-Net is designed for biomedical segmentation [45]. U-Net consists of two paths forming a U-shape: a contraction and an expansion path. The contraction path consists of four blocks, where each block has two convolutions, two ReLUs, and one max pooling layer. The expansion path has also four blocks; however, upsampling is used rather than downsampling, and concatenation followed by regular convolution operations. The contraction path enables the network to learn context, whereas the expansion path preserves the spatial information. This design is very important for our work. It allows retrieving high resolution feature maps and preserving image details such as cell boundaries.

In Faster R-CNN [39], a convolutional neural network like VGG-16 is typically used as the feature extraction backbone. VGG-16 is 16 layers deep and has been optimized to classify images with 1000 classes for the ImageNet competition (ILSVR 2014). However, in our case, we have just a two class task to identify RBC versus background; additionally our cells are small and have a relatively homogeneous appearance and texture. So instead of using a deep backbone like VGG-16 or ResNet-50 for Faster R-CNN, we designed a *customized* CNN backbone with fewer layers for cell feature extraction consisting of nine layers: one input layer, two convolutional plus ReLU layers, followed by one pooling layer, then two fully connected layers, a softmax layer and a final classification layer. For training RBCNet, we use labeled fixed size tiles for both U-Net and the customized Faster R-CNN, $256 \times 256$ and $128 \times 228$ respectively, for fast training and obtained a robust learned RBCNet model. The details of our cross validation experiments are in Section II-A.

**Fig. 3.**
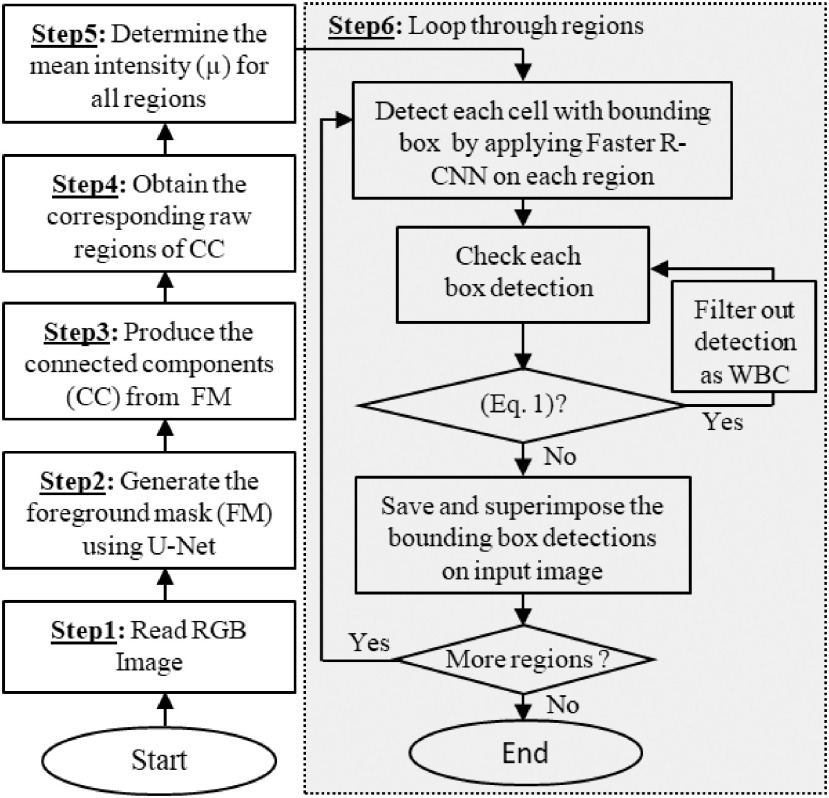
RBCNet inference flowchart starts with reading an input image, applying U-Net and Faster R-CNN, and ends with saving all detected cell bounding boxes.

**Inference Step:** Using the trained models of U-Net and Faster R-CNN, we proposed RBCNet, a robust pipeline for cell segmentation shown in Fig. 3. The flowchart begins with reading the input image and ends with detecting all RBCs. The shaded box describes Step 6 which combines the information from U-Net with Faster R-CNN inferencing. Step 6 loops over each extracted connected component (image object mask region) and applies the trained deep architecture of Faster R-CNN to localize each cell using a detection bounding box. See Figure 6 for some examples.

**Fig. 4.**
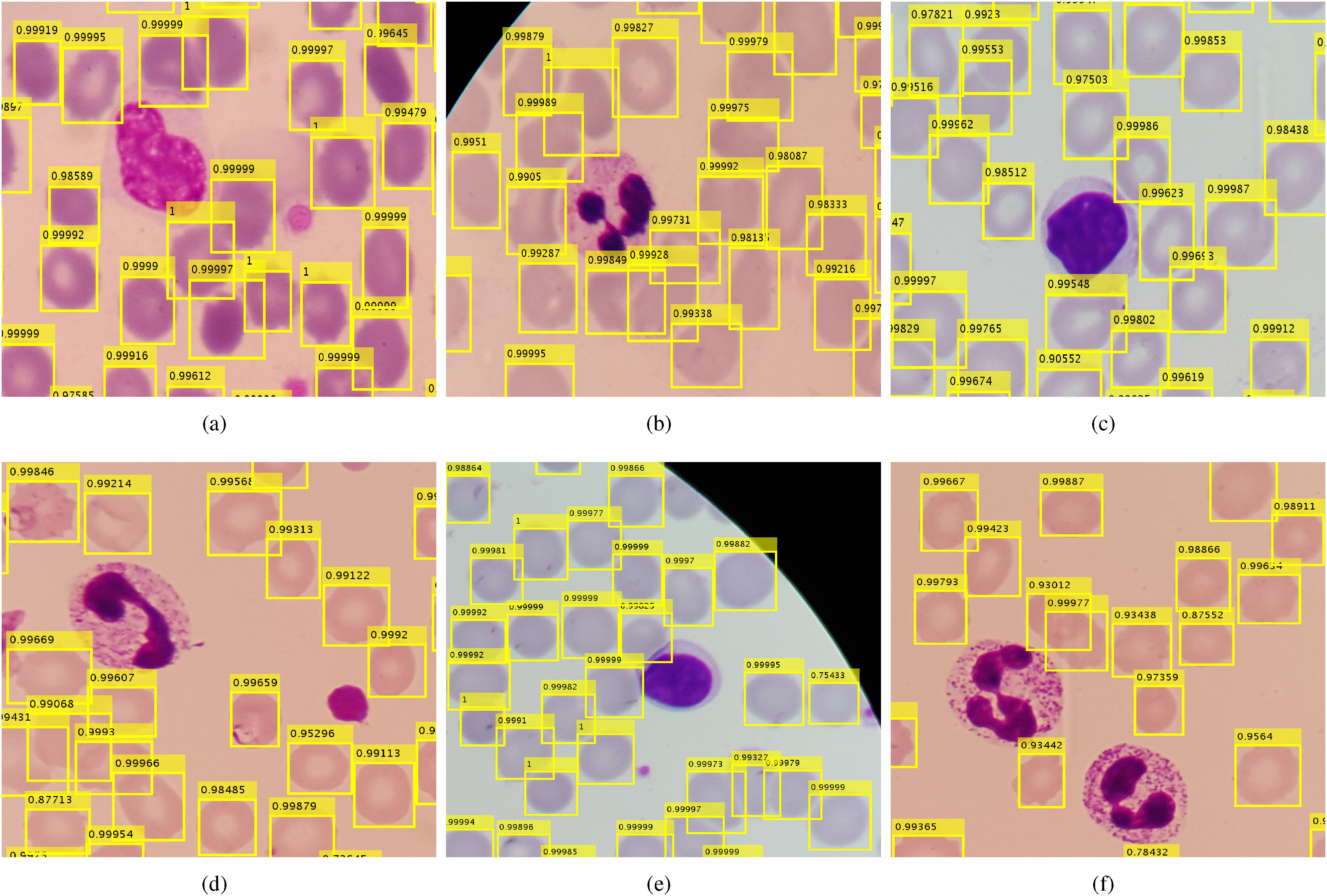
Examples of our RBCNet cell detection results showing RBCs and WBCs in different colors, shapes, and illumination conditions (cropped ROIs at original resolution). Our proposed RBCNet pipeline successfully detects RBCs and filters out WBCs in all these cases.

**Fig. 5.**
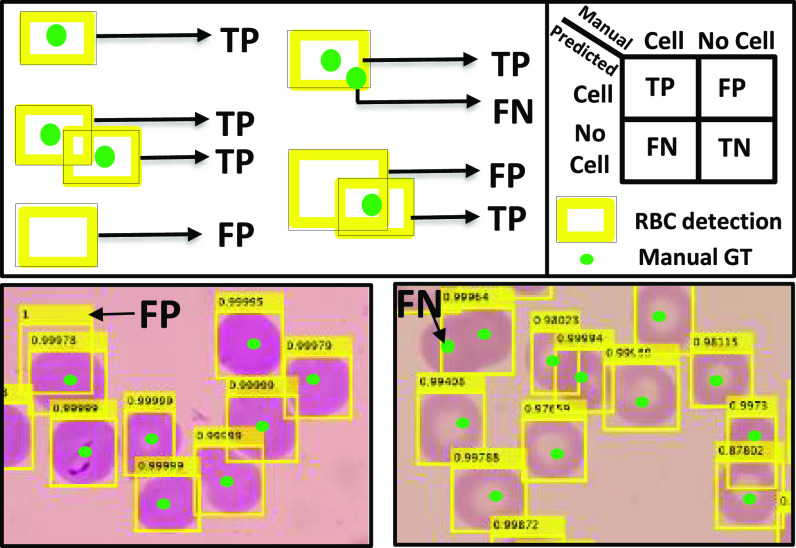
Evaluating detection accuracy for counting RBCs using one-to-one matching assessment with the confusion matrix shown in the upper right. Upper left figure shows examples of TP, FP, and FN. For point correspondences we do not use specificity or TN rate as most of the image pixels are TN. In the bottom row the yellow bounding boxes are RBCNet algorithm detections and the green points are the expert's manual ground-truth point annotations.

**Fig. 6.**
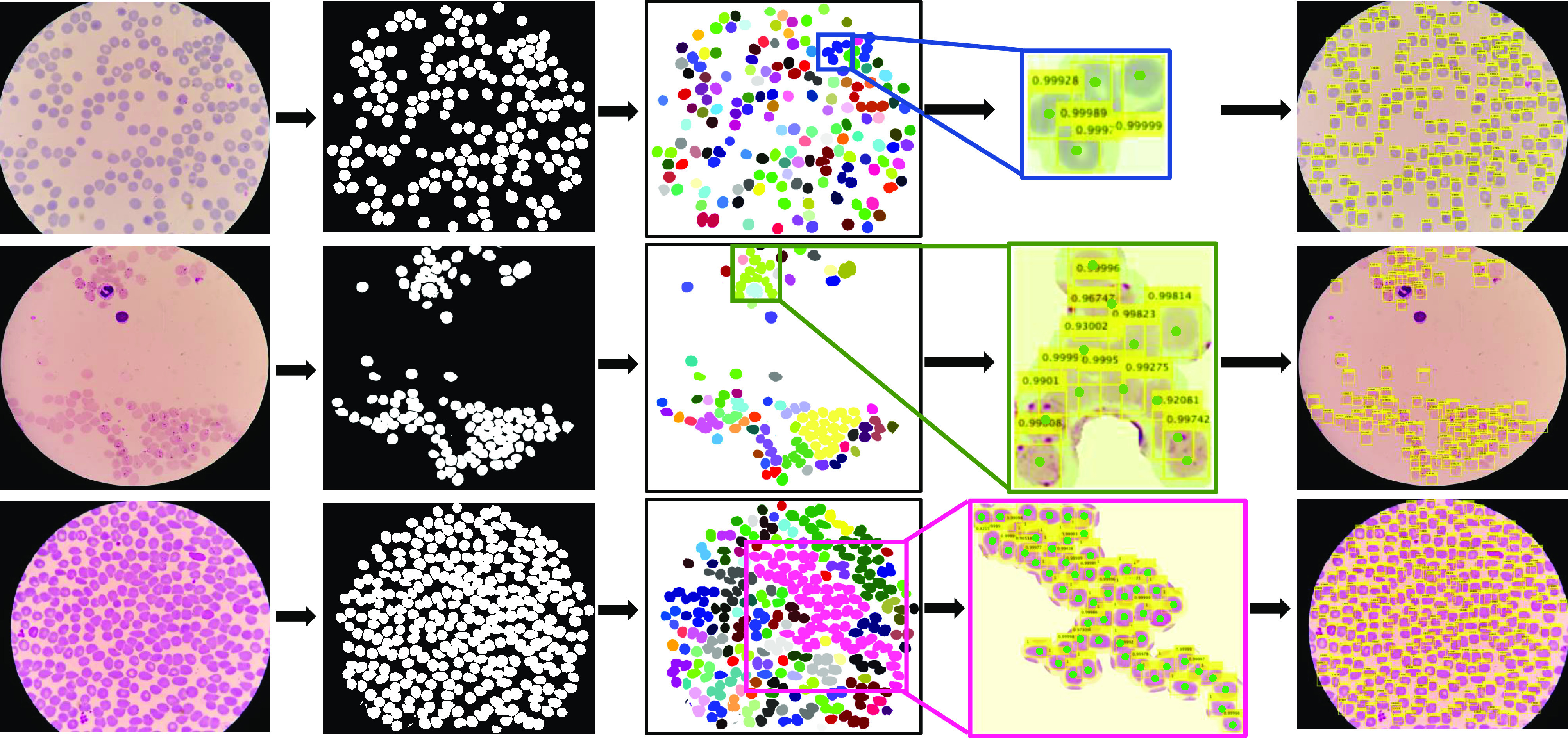
Three examples of cell clumps of different sizes for which our dual deep network architecture pipeline **RBCNet** successfully identifies individual cells. The examples show the connected components generated from the U-Net foreground mask and the bounding boxes of individual cells detected by our Faster R-CNN model. The first column shows the field of view for the raw image, the second column shows the binary mask produced by U-Net, and the third column shows the connected component labeling for U-Net's binary mask. In the fourth column are zoomed-in examples of connected components for which Faster R-CNN detects all cells. Green dots represent the gold standard ground- truth (GT) annotation. The last column visualizes all bounding boxes for all cell clumps superimposed on the raw image. Combination of U-Net training using tiles along with connected component labeling for the output mask enables cells and cell clusters (i.e. touching or overlapping cells) to be accurately detected and segmented across tile boundaries. The cell clusters identified by U-Net typically have smooth borders and very little fragmentation.

**Post-Processing Step:** Our algorithm filters out any bounding box satisfying Eq. 1 as a leukocyte or white blood cell (WBC) and saves all remaining bounding boxes for evaluation.

\begin{equation*}
\sum _{c=0}^{n} f(\mu -\epsilon -I_c) > T \tag{1}
\end{equation*}

where

\begin{equation*}
f(x) = \left\lbrace \begin{array}{lll}0 &\text{ if } x \leq 0 &\text{RBC pixel} \\
1 &\text{ otherwise},& \text{WBC pixel} \end{array} \right.
\end{equation*}

and $n$ is the number of foreground pixels within each detection, $I_c$ is the intensity value for each pixel, $T$ is a predefined threshold, and $\epsilon$ is a heuristic estimate on how much darker a WBC is, on average, compared to the mean intensity $\mu$. WBCs are up to three times larger than RBCs and are usually darker than RBCs. We found that thresholding on the number of dark pixels typically associated with WBC detections using Eq. 1 is a good solution. According to Eq. 1, we detect and filter out any bounding box as a WBC object if it contains more than $T$ pixels of sufficient darkness. See Fig. 4 for some examples of patches that have both RBCs and WBCs, and where our algorithm filters out all WBCs successfully.

### A. Cross Validation

In our work, we evaluate both the polygon set and the point set. To evaluate our RBCNet on our polygon set (33 patients), we apply 11-fold cross validation on patient level. In each fold, we use the imagery of 30 patients (150 images) for training and exclude three patients (15 images) for testing. To evaluate the point set (160 patients), we train all the images in the polygon set. Table I illustrates the data statistics of our experiments. All experiments ran for 20 epochs on an Nvidia GeForce GTX 1080Ti GPU. For U-Net and Faster R-CNN, we use implementations provided by the Neural network toolbox in MATLAB [46].

**TABLE I:** Experimental Setup for Training our Dual Deep Learning Architecture

Data Set	# of Patients	# of Images	# of Cells	Cross Validation	Training (#Images/Fold) (#Patients/fold)	Testing (#Images/Fold) (#Patients/Fold)
Polygon Set	33	165	34,226	11 - Fold	150 images from 30 patients	15 images from 3 patients
Point Set	160	800	162,443	Train all images in polygon set	165 images from 33 patients	800 images from 160 patients

During training the Faster R-CNN stage, the input images are rescaled from $5312 \times 2988$ to 0.3 of their original size. Using $128 \times 228$ rectangular tiles, the smaller image size of $1594 \times 896$ results in tiles with a higher density of RBCs (smaller cell sizes) within each tile. Specifically, each tile has about 7 to 15 RBCs and there are about 49 tiles per image. However, we only consider the tiles within the field of view and exclude all of the black background tiles. So, the number of tiles used per image is about 25, which leads to a training set of 3750 tiles (25 tiles/image $\times 150$ images) used in each fold. We use a small subset of the training tiles for validation (about 100 tiles) to monitor the accuracy and loss during training, which determines if more epochs or training samples are needed. For evaluating on our point set (160 patients with 800 images), we first train on all the images in our polygon set (165 images from 33 patients) by generating a training set with 4125 tiles (25 tiles/image $\times 165$ images).

Faster R-CNN training happens in four phases: Phase 1 and 2 train the region proposal and detection networks. In the last two phases a single combined network is trained for detection. We use a higher learning rate in Phase 1 and 2, equal to 0.001, then for the last two phases, we decrease the learning rate to 0.0001 because the last two phases are just to fine tune the network weights. The network is trained from scratch. The number of layers and parameters have been selected after extensive trials for several configurations. We train U-Net in a similar fashion (i.e. same cross validation scheme). Our objective is to learn a robust U-Net model for predicting accurate foreground masks because our proposed approach depends on this mask during the inference stage. We generate training data by randomly cropping 100 patches per image, with dimensions of $256 \times 256$, and by augmenting them through random rotation, reflection, and scaling to increase the number of examples for a more robust training. In this way, we create eight additional patches for each single patch, which increases our training set to $120{,}000$ patches $(150 \times 100 \times 8)$ for training each fold to evaluate the polygon set. Similarly, to evaluate the point set, we augment the training data of the polygon set to create a larger training set consisting of $132{,}000$ patches $(165 \times 100 \times 8)$. On this larger training data, U-Net needs 20 epochs to converge. We trained the network using stochastic gradient descent with momentum (SGDM) optimization. The bias term of all convolutional layers is initialized to zero and convolution layer weights in the encoder and decoder sub-networks are initialized using the ‘He’ weight initialization method [47]. We accelerate the training by setting a high learning rate equal to 0.05; however, to prevent the gradients of the network from exploding, we enable a gradient clipping threshold equal to 0.05, and clip the gradients if their L2-norm exceeds the given threshold.

### B. Performance Metrics

We evaluate the red blood cell detection algorithms based on a one-to-one matching between point annotations in our ground-truth, which represent individual cells identified by an expert microscopist, and the detected cells. To evaluate our cell detection quantitatively, we apply the following evaluation scheme, which is also visualized in Fig. 5:

1) If a detected bounding box contains just one point in the labeled data, consider it a true positive (TP).
2) If the bounding box contains more than one point, consider the one which is closest to the center of the bounding box as a TP. Remaining points are either TP if there are other bounding boxes containing each of them, otherwise are missed detections so false negatives (FN).
3) If any point is not contained in a box, label it as a false negative (FN).
4) If a bounding box does not contain any point, then label this detection as a false positive (FP).

Our evaluation considers the standard performance metrics Recall, Precision, and F1-measure for evaluating our RBCNet cell detection results and comparing them to the expert gold standard. Recall is a statistical measure used to quantify how well an algorithm detects objects, or cells in our case. While Precision assesses how robust it is in avoiding false detections,

\begin{equation*}
Recall= \frac{TP}{TP+FN},\quad Precision= \frac{TP}{TP+FP}.
\end{equation*}

The F1 measure combines Precision and Recall, using the harmonic mean between the two,

\begin{equation*}
F1\text {-}measure = 2\cdot \frac{Recall \cdot Precision}{Recall+Precision}.
\end{equation*}

## III. Experimental Evaluation of RBCNet

In this section, we first address the various methods and strategies that we have used to detect the cells in our data set in III-A, then we describe and discuss the results in III-B.

### A. Comparisons and Testing Strategies

Several methods and experiments have been tested and conducted to detect cells in our thin smear data set including our proposed RBCNet. We divide them into four groups:

*1) Traditional methods:* We compare our algorithm results with two state-of-the-art traditional methods in cell detection: Active contour [18], [19] and Watershed [17]. These two algorithms are the core of most existing papers to localize and separate cells, as we discussed in Section I-A. They are considered superior for their efficiency to detect cells based on specific criteria and without the need to train and validate deep networks or acquiring GT labeling. However, these methods may fail for images with extreme and challenging conditions. Furthermore, the optimization of hyper-parameters for Active contours can be computationally expensive.

*2) Instance segmentation DL methods:* Instance segmentation methods provide automatic delineation for each object in the image on pixel-level. These methods may fail to produce a robust separation with images that are too dense with overlapping small objects such as our cell images. They can succeed in separating some cells but leave others as clumps of cells. We utilize three popular deep learning architectures: SegNet [48], U-Net [42], and DeepLabV3+ [49].

*3) Object detection DL methods:* We utilize four state of the art object detection networks: Faster R-CNN [39], You Look Only Once (YOLO) [50], Single Shot Detection (SSD) [51], and Mask R-CNN [52]. They are all trained from scratch using the same number of tiles and share the same parameters. All networks can accept a small or regular image size dimension ($\leq$ 600), however, they cannot work with extremely large image size dimensions. The only solution is to train the networks using tiles. However, tiling can be problematic in the inference stage because of cell fragmentation, specifically, when object density is high. We follow four strategies in the testing stage: testing using the full image after resizing it to smaller dimensions, testing with non-overlapping tiles, testing with overlapping-tiles, and finally testing with overlapping-tiles with non maximum suppression (NMS) to remove replicated bounding boxes for a cell. NMS has been widely used as post processing for many computer vision applications, including object detection [53], [54]. NMS keeps the bounding boxes with a high confidence score and eliminates the nearby windows with lower confidence scores. The testing scores for all testing strategies are discussed in Section III-B. Our proposed dual network architecture addresses all the limitations produced by the tiling process and considers a different strategy in the testing stage.

*4) Proposed dual deep learning networks:* Our proposed dual networks solve the detection limitations for the tiling process in the testing stage for dense and large images. They detect tiles with cell clumps, obtained by an instance segmentation DL method, which are not prone to cell fragmentation. Furthermore, better cell delineation, resulting from Instance segmentation methods, leads to smaller cell clumps and more accurate detection results. We use three combined networks: SegNet+Faster R-CNN, U-Net+YOLO, and our proposed RBCNet (U-Net+Faster R-CNN). Although several other combinations are possible, we only try these three combinations for several reasons discussed in Section III-B.

### B. Experimental Results with Discussion

Tables II and III display the experimental results for the experiments conducted using the methods described in Section III-A.

**TABLE II:** Segmentation Accuracy for our RBC Polygon Set, Using 11-fold Cross Validation. For Each Experiment, the Training Set Contains Tiles From 150 Images, and the Test Set Contains 15 Images. We Conducted t-Tests Using the F1-Measure Between our Proposed RBCNet Dual Network (U-Net+Faster RCNN) and Other Methods. All p-Values are < 0.001, Indicating That the Differences Between Groups are Statistically Significant

Method $\backslash$ Evaluation Metrics	F1-Measure $\pm SD$	Precision $\pm SD$	Recall $\pm SD$
**Traditional methods**			
Watershed [17]	$94.62 \pm 5.62$	$96.50 \pm 2.01$	$93.34\pm 9.28$
Active contour [18], [19]	$96.33 \pm 1.97$	$95.64 \pm 2.31$	$97.11\pm 3.02$
**Instance segmentation deep learning methods**			
SegNet [48]	$55.97 \pm 23.1$	$84.20 \pm 10.2$	$45.45 \pm 24.3$
U-Net [42]	$87.06 \pm 13.3$	$93.48 \pm 4.5$	$83.33 \pm 17.6$
DeepLab v3+ [49]	$74.59 \pm 24.4$	$91.97 \pm 3.4$	$68.37\pm 28.2$
**Object detection deep learning methods**			
Faster R-CNN [39] on overlapping-tiles + NMS	$91.19\pm 2.51$	$90.39\pm 3.85$	$92.47\pm 3.34$
Yolo [50] on overlapping-tiles + NMS	$96.13 \pm 1.72$	$97.09 \pm 1.79$	$95.27\pm 3.19$
SSD [51] on overlapping-tiles + NMS	$87.26 \pm 2.52$	$89.63 \pm 3.06$	$85.18 \pm 4.17$
Mask R-CNN [52] on overlapping-tiles + NMS	$95.62 +1.57$	$98.39\pm 1.12$	$93.05 +2.82$
**Proposed dual deep learning networks**			
SegNet + Faster R-CNN	$96.80\pm 2.03$	$ 96.16 \pm 2.03$	$97.53 \pm 3.37$
U-Net + YOLO	$96.07 \pm 6.42$	${\bf 98.58}\pm 1.29$	$94.23\pm 9.36$
**RBCNet** (U-Net + Faster R-CNN)	${\bf 97.76} \pm 1.71$	$97.51\pm 1.58$	${\bf 98.07}\pm 2.97$

**TABLE III:** Detection Accuracy for Our RBC Point Set. All Training Data of the Polygon Set (165 Images) has Been Used to Generate the Training Model to Test 800 Images From 160 Patients. The t-Tests Between Our Proposed Dual RBCNet and Other Methods Have P-Values < 0.001, Indicating That the Differences Between Groups are Statistically Significant

Method $\backslash$ Evaluation Metrics	F1-Measure $\pm SD$	Precision $\pm SD$	Recall $\pm SD$
**Traditional methods**			
Watershed [17]	$94.31 \pm 9.07$	$95.56 \pm 7.84$	$93.51 \pm 11.26$
Active contour[18], [19]	$95.66 \pm 7.98$	$94.99 \pm 7.87$	$96.44 \pm 8.60$
**Instance segmentation deep learning methods**			
SegNet [48]	$71.54 \pm 19.1$	$92.27 \pm 5.4$	$61.46 \pm 22.4$
U-Net [42]	$91.93 \pm 9.05$	$ 95.41 \pm 2.71$	$89.72 \pm 12.8$
DeepLab v3+ [49]	$79.44 \pm 19.4$	$92.10 \pm 3.6$	$73.75 \pm 23.9$
**Object detection deep learning methods**			
Faster R-CNN [39] on overlapping-tiles + NMS	$91.73\pm 5.23$	$88.58\pm 7.07$	$95.84\pm 6.68$
Yolo [50] on overlapping-tiles + NMS	$96.23\pm 1.42$	$97.86\pm 1.36$	$94.69\pm 2.23$
SSD [51] on overlapping-tiles + NMS	$87.31\pm 2.39$	$89.45\pm 3.24$	$ 85.41\pm 3.58$
Mask R-CNN [52] on overlapping-tiles + NMS	$95.58\pm 1.64$	$98.05 \pm 1.25$	$93.28 \pm 2.83$
**Proposed dual deep learning networks**			
SegNet + Faster R-CNN	$97.52 \pm 1.49$	$97.23 \pm 1.58$	$97.86 \pm 2.52$
U-Net + YOLO	$96.00 \pm 5.86$	${\bf 98.2}\pm 1.35$	$94.38\pm 8.99$
**RBCNet** (U-Net + Faster R-CNN)	${\bf 97.94}\pm 1.32$	$97.54 \pm 1.44$	$ {\bf 98.39} \pm 2.24$

**Fig. 7.**
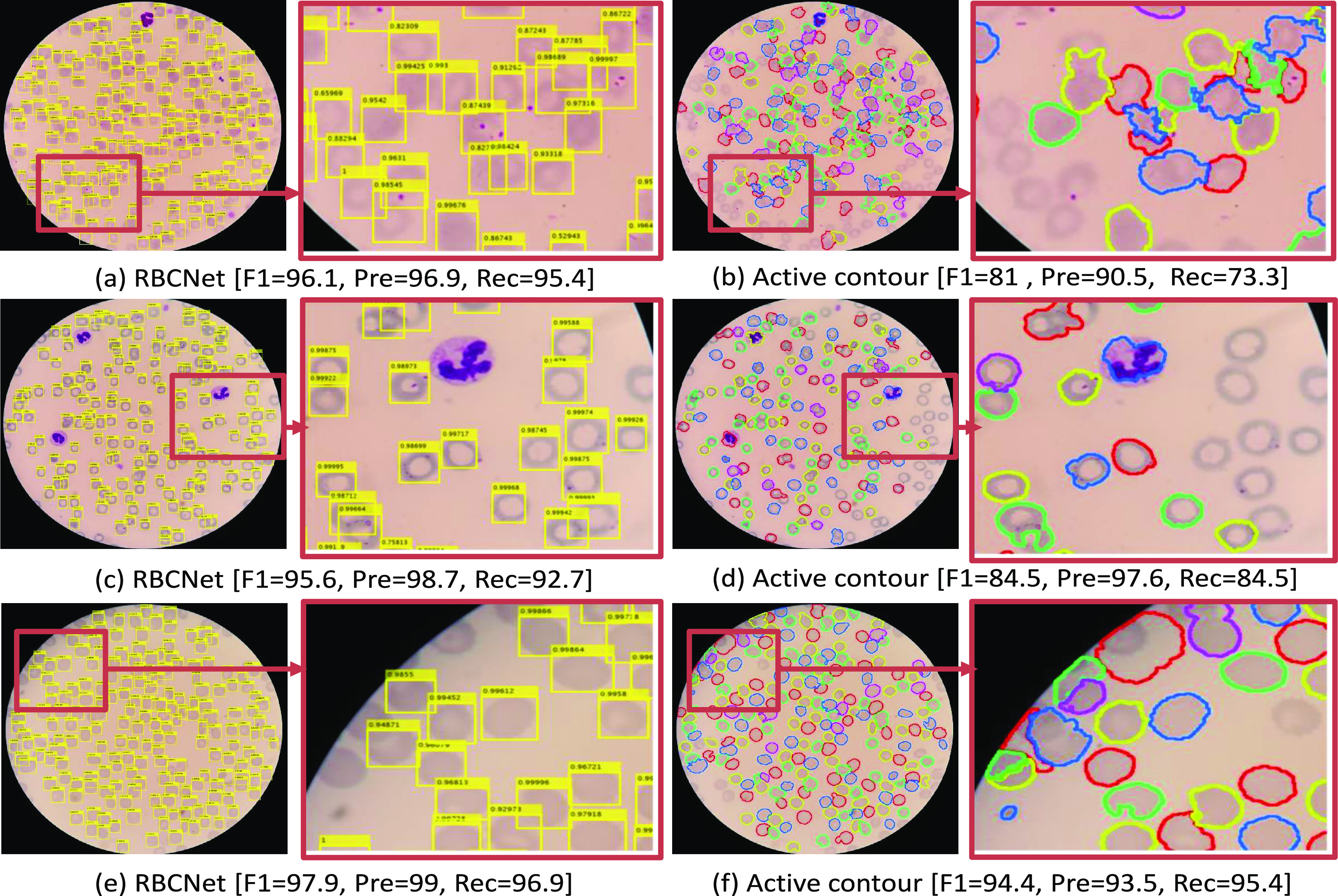
Comparison between our RBCNet pipeline results and one of the very popular traditional methods in the literature for cell detection, based on Active contours [18], [19]. Panels a,c, and e show the results of our proposed processing pipeline applied to several sample images. Panels b,d, and f show examples for the traditional Active contours method.

*1) Watershed [17] and Active contour [18], [19]:* Row 3 and 4 in Tables II and III show the quantitative results for Watershed and Active contour methods. Traditional methods may work very well for many images, but can fail suddenly when encountering challenging conditions because they depend on specific criteria, such as intensity or energy. This also becomes evident when comparing the differences between standard deviations (SDs). For example, in Table III, the SD for Watershed and Active contour is around six times higher than the SD of the results produced by our method. Fig. 7 shows several examples where our prediction results differ from the predictions produced by Active contours. Our pipeline works consistently well for all regions, whereas Active contour results suffer from either under- or over-segmentation, which typically happens in regions with challenging conditions such as low contrast or illumination variation. RBCNet performs more robustly in the boundary regions, where cells are often only partly visible. For example, in sub-figure (f) of Figure 7, Active contour produces over-segmentation for partly visible cells in this region, whereas our pipeline predicts only the fully visible cells, which is a desirable feature closer to the human expert strategy. Comparing our RBCNet to traditional methods (Active contour and Watershed), the relative improvement is larger for the point set than for the polygon set. This is because the point set contains data from about five times more patients than the polygon set. Having more patients leads to a higher variability in terms of illumination, shape, and cell density, which poses problems to traditional approaches.

*2) Instance segmentation DL methods:* Tables II and III show the evaluation scores of SegNet [48], U-Net [42], and DeepLabV3+[49]. U-Net performs better than other methods with F1-measure equal to 87% for the polygon set and 92% for the point set. For this reason, we choose U-Net to be part of our final dual network RBCNet.

*3) Object detection networks with different tiling strategies:* We discuss here the four tiling strategies mentioned in III-A that are regularly used in the inference stage for object detection networks. We use Faster R-CNN for our analysis, see Table IV. Faster R-CNN has the advantage that it can accept any input size. Once the model is trained, we can apply it to both the full image or image tiles. We test our trained model on the full image as a first strategy because this is faster and easier. However, the results were not promising, as can be seen in Table IV. In particular, the recall is very low for this straightforward approach. Recall is equal to 66% for the polygon set and 69% for the point set. This is because the full image has a different size compared to the tiles used for training. The ratio of image to cell size is much larger than the ratio of tile size to cell size.

**TABLE IV:** Detection Accuracy for Our RBC Polygon and Point Sets for Different Tiling Strategies Using Faster R-CNN [51]

Method$\backslash$ Evaluation Metrics	F1-Measure $\pm SD$	Precision $\pm SD$	Recall $\pm SD$
**Polygon set (33 patients /165 images)**			
Full image	$76.33\pm 12.78$	$96.83\pm 1.59$	$66.13\pm 15.26$
Non-overlapping tiles	$84.21\pm 3.76$	$93.64 \pm 2.27$	$77.11 \pm 6.39$
Overlapping-tiles	$72.32\pm 2.59$	$59.27\pm 4.13$	$93.65\pm 3.03$
Overlapping-tiles + NMS	$91.19\pm 2.51$	$90.39\pm 3.85$	$92.47\pm 3.34$
**RBCNet** (U-Net + Faster R-CNN)	${\bf 97.76} \pm 1.71$	${\bf 97.51}\pm 1.58$	${\bf 98.07}\pm 2.97$
**Point set (160 patients/ 800 images)**			
Full image	$79.16\pm 13.11$	$95.57\pm 2.39$	$69.48\pm 17.28$
Non-overlapping tiles	$85.55 \pm 6.27$	$92.56\pm 3.76$	$80.46\pm 10.31$
Overlapping-tiles	$71.88\pm 4.69$	$57.95\pm 6.45$	$96.19\pm 6.57$
Overlapping-tiles + NMS	$91.73\pm 5.23$	$88.58\pm 7.07$	$95.84\pm 6.68$
**RBCNet** (U-Net + Faster R-CNN)	$ {\bf 97.94} \pm 1.32$	$ {\bf 97.54} \pm 1.44$	$ {\bf 98.39} \pm 2.24$

A second strategy is to implement a tile-based inference stage, which is consistent with our tile-based training. Table IV shows the performance evaluation for Faster R-CNN on non-overlapping tiles. It is noticeable that the recall is still low because some cells are not detected, as they have been cut off by the tiling process.

Therefore, a third strategy is to use overlapping tiles with an overlap ratio of 50%. In Table IV, Faster R-CNN on overlapping tiles shows our evaluation results for this strategy for both polygon and point set. We achieve a relatively high recall of 93.65% and 96.19%, respectively. However, using overlapping tiles leads to duplicate detection of some cells. For this reason, this strategy has the worst precision compared to other approaches in both tables, 59.27% and 57.95%. NMS leads to relatively good evaluation results, as shown in Table IV for both polygon and point set. Applying NMS increases the precision by 30%, with only a moderate loss in recall around 1%. We applied NMS with an overlap ratio of 0.5, which means it filters out all the bounding boxes that overlap more than 50%. Increasing the overlap ratio to higher values would decrease the recall because RBCs can overlap in dense cell clusters. According to our results listed in Table IV, Faster R-CNN on overlapping-tiles with NMS produces the best result compared to other tiling strategies. This has encouraged us to consider this strategy to produce good results for other object detection networks in Tables II and III.

Figure 9 illustrates the output of Faster R-CNN using these four different strategies and shows how our RBCNet with U-Net masks performs significantly better than other inference approaches. Testing on full images and testing with non-overlapping tiles lead to a low recall as shown in panels [a and f] and panels [b and g], whereas testing with overlapping tiles leads to a low precision because some cells are predicted twice as shown in panels [c and h]. Applying NMS with an overlap ratio higher than 0.5 can remove some of the duplicate predictions; however, NMS cannot remove all the extra bounding boxes generated, see panels [d and i]. Our RBCNet in the last column provides the best detection performance. Red dots correspond to the cells that have not been detected (FN) while blue dots represent duplicate detections (FP).

**Fig. 8.**
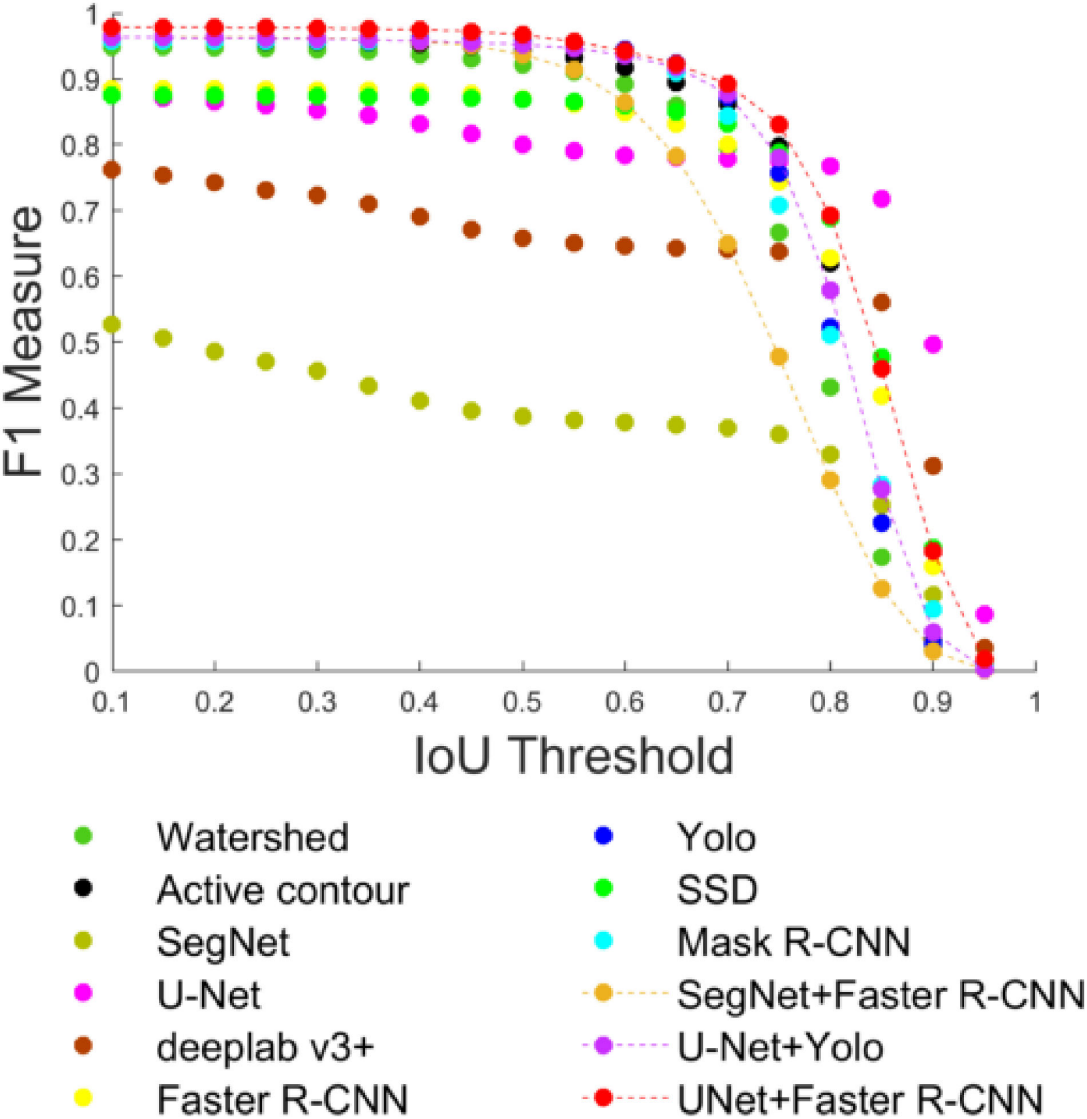
F1-measure versus IoU threshold for all experiments using the polygon set and 34,213 GT cells. The plot shows how the F1-measure decreases as the IoU threshold or overlap ratio is increased.

**Fig. 9.**
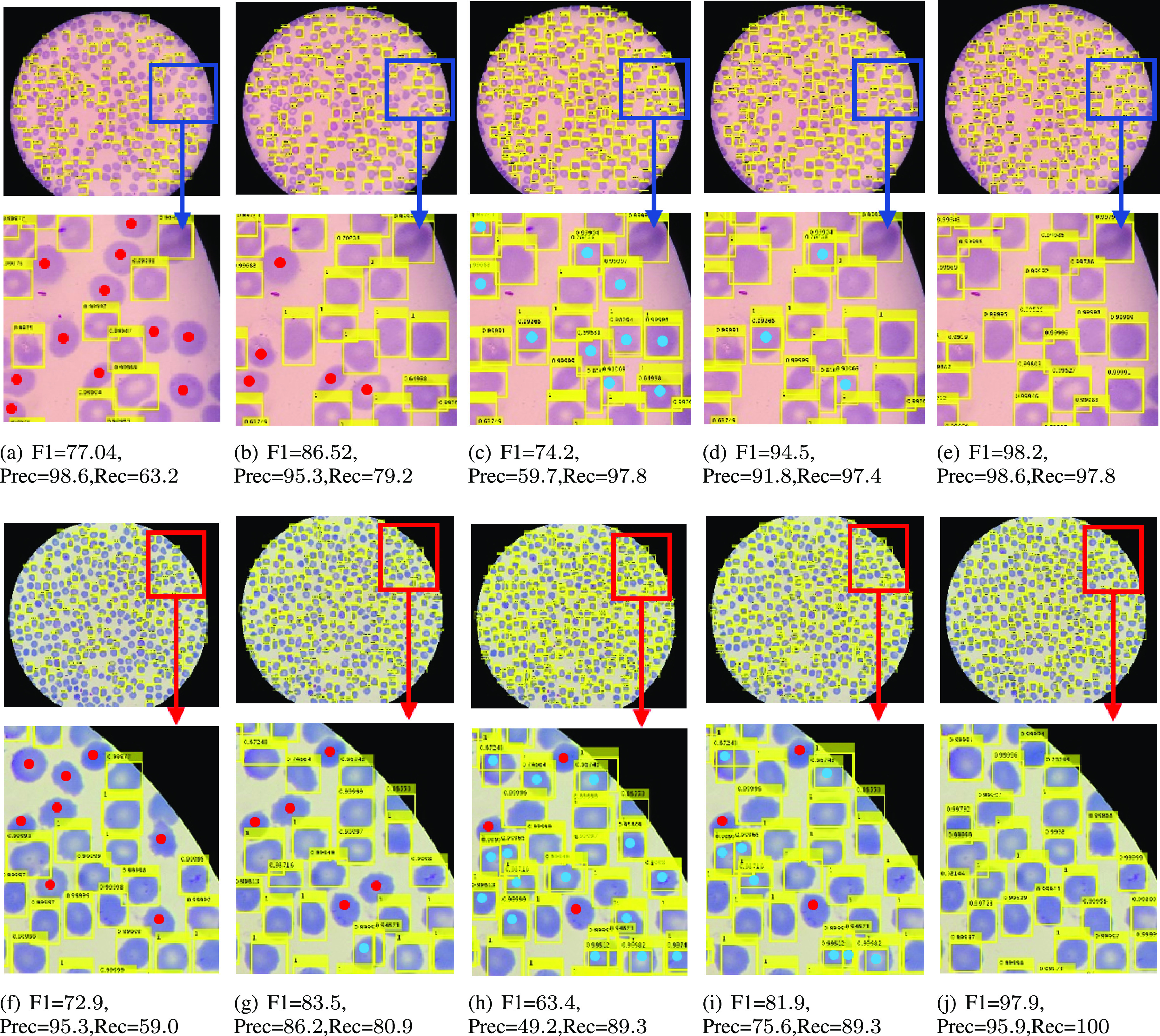
Output of Faster R-CNN for different input configurations: First column (a, f) shows Faster R-CNN applied to the full image, second column (b, g) shows Faster R-CNN results for non-overlapping tiles, third column (c, h) are Faster R-CNN results on overlapping tiles, fourth column (d, i) contains results with overlapping tiles and non-maximum suppression, and last column (e, j) shows results for our proposed method. Red dots $\color{red}{{\bullet}}$ represent false negatives and blue dots $\color{blue}{{\bullet}}$ represent false positives. Our results in the last column provide a better F1 measure in all cases compared to other inference schemes.

*4) Our proposed dual deep network architecture:* The first combined architecture that we have tried is U-Net+YOLO as those two networks produce the highest DL F1-measure in both Tables II and III. However, we found that YOLO is sensitive to tile size and only responds well for tiles that have a similar dimension as the training tiles. It does not respond well for tiles with large cell clumps. Although the recall did not improve, it is noticeable that the precision increased because our method tested tiles with full clumps; not tiles with fragmented small cell objects. As a second trial, we choose Faster R-CNN, as it has the highest recall/detection in Table III. Note that we did not choose Mask R-CNN because it needs a larger set of polygon/segmentation mask training data, which is expensive and its acquisition takes longer. Mask R-CNN is also much slower than Faster R-CNN. Another advantage of Faster R-CNN is that it is more versatile and automatically adapts to different tile sizes. It accepts clumps/clusters with different numbers of cells or tile sizes for inference.

Our proposed dual deep network architecture RBCNet takes advantage of U-Net to provide initial foreground masks as input to Faster R-CNN for cell identification. This dual architecture has the following advantages:

U-Net can provide a foreground mask with the connected components of the corresponding image regions as input to Faster R-CNN. Using connected components as input to Faster R-CNN has the advantage that no cells will be cut-off, which happens in tile-based approaches. This results in higher true positive and lower false positive rates.

Touching and overlapping cells are usually identified as a single clump by U-Net. Faster R-CNN is able to identify individual cells within clumps accurately and rarely produces a false detection in the background, even in places with illumination artifacts and other challenging conditions, because it is guided by the U-Net foreground mask.

U-Net preserves the spatial details lost during down-sampling by concatenating the cropped feature maps with the corresponding maps through up-sampling. Preserving spatial details of RBCs improves segmentation because it leads to more robust candidates for Faster R-CNN. To illustrate this, replacing U-Net by SegNet [48] shows the effects of preserving details through concatenation rather than just transferring max pooling indices to the decoder. Hence, SegNet does not preserve important neighboring information like U-Net.

For all these combinations, we show the results in the last three rows of Tables II and III. Our proposed dual deep learning architecture RBCNet, using U-Net foreground mask with Faster R-CNN, outperformed all other methods evaluated with the F1-measure, with a very low standard deviation (SD). Tables II and III show our experimental results, where numbers in bold represent our best results. For the polygon set, we achieve an F1-measure, precision, and recall of 97.76%, 97.51%, and 98.07%, respectively. For our point set, we achieve 97.94%, 97.54%, and 98.39% correspondingly.

Fig. 8 shows the F1-measure for the polygon set plotted versus the Intersection over Union (IoU) metric threshold between detected cells and the corresponding GTs. Although, we cannot produce the same plot for the point set because we do not have GT polygon annotations to determine the overlap, this plot gives us an idea about the robustness and stability of our architecture. It shows that the F1-measure decreases while the IoU overlap threshold increases. This is because the number of TPs decreases, and more cells become FPs, since detections have less than the required overlap with GT. Our dual RBCNet architecture is relatively stable against this IoU metric. F1-measure remains over 90% until the IoU overlap requirement becomes larger than 0.75. It is also noticeable that U-Net and Deeplab v3+ produce a relatively more stable F1-measure for IoU between 0.8 and 1. However, these two methods have an overall lower F1-measure.

We also apply a t-Test to determine whether there is a significant difference between the means of our RBCNet and other methods in terms of F1-measure. We compute p-values that are less than 0.001 for all the experiments, which shows that the differences are statistically significant.

For a straightforward implementation on a regular PC and without a GPU, the processing time varies between 20 and 60 seconds per image depending on the cell density. However, our method can be parallelized because RBC clusters can be processed independently, and in parallel. We estimate that this would reduce the total processing time significantly.

## IV. Conclusion

Our dual deep learning architecture RBCNet, which combines U-Net with Faster R-CNN, provides a robust solution for detecting RBCs in blood smear images characterized by a small ratio of cell object size to image size. For automated malaria screening, we tested our proposed pipeline on 965 images from different patients to detect single RBCs and to segment overlapping RBCs in cell clusters. By applying Faster R-CNN on a foreground mask produced by U-Net, we are able to outperform traditional cell detection methods, instance segmentation deep learning methods, and object detection deep learning methods. Our cell detection implements a crucial step towards automated malaria diagnosis. Future work will combine our cell detection pipeline with a cell classifier to differentiate between infected and uninfected cells.
